# Evaluation of the Accuracy of Bathymetry on the Nearshore Coastlines of Western Korea from Satellite Altimetry, Multi-Beam, and Airborne Bathymetric LiDAR

**DOI:** 10.3390/s18092926

**Published:** 2018-09-03

**Authors:** Yeon Yeu, Jurng-Jae Yee, Hong Sik Yun, Kwang Bae Kim

**Affiliations:** 1 National Research Center for Disaster-free and Safety Ocean City Construction, Dong-a University, Busan 49315, Korea; yeu@dau.ac.kr; 2 Department of Architectural Engineering, Dong-a University, Busan 49315, Korea; jjyee@dau.ac.kr; 3 School of Civil, Architectural and Environmental System Engineering, Sungkyunkwan University, Suwon 16419, Korea; yoonhs@skku.edu

**Keywords:** bathymetric mapping, satellite altimetry-derived gravity anomalies, multi-beam, airborne bathymetric LiDAR

## Abstract

Bathymetric mapping is traditionally implemented using shipborne single-beam, multi-beam, and side-scan sonar sensors. Procuring bathymetric data near coastlines using shipborne sensors is difficult, however, this type of data is important for maritime safety, marine territory management, climate change monitoring, and disaster preparedness. In recent years, the bathymetric light detection and ranging (LiDAR) technique has been tried to get seamless geospatial data from land to submarine topography. This paper evaluated the accuracy of bathymetry generated near coastlines from satellite altimetry-derived gravity anomalies and multi-beam bathymetry using a tuning density contrast of 5000 kg/m^3^ determined by the gravity-geologic method. Comparing with the predicted bathymetry of using only multi-beam depth data, 78% root mean square error from both multi-beam and airborne bathymetric LiDAR was improved in shallow waters of nearshore coastlines of the western Korea. As a result, the satellite-derived bathymetry estimated from the multi-beam and the airborne bathymetric LiDAR was enhanced to the accuracy of about 0.2 m.

## 1. Introduction

Coastlines (or shorelines) have been investigated as one of the most important features on the Earth’s surface due to coastal erosion and flooding, adaptation to climate changes, coastal protection, environmental impact assessment, disaster management, and sustainable development. The information about coastlines and their changes is essential for coastal zone environmental management. In the past, coastlines were detected through ground surveying to generate coastline maps. As the technology has been developed, new methods were used to map coastlines. Aerial photographs were used for delineating and monitoring coastal erosion over a long coast in an efficient and economical way [1,2]. Global positioning system (GPS) is a useful technique for a spatial and temporal analysis. The beach face profile was observed by GPS and aerial photographs to examine shoreline mobility and sediment budget on a sandy coast over several years [3]. Coastline variations were determined by the real-time kinematic GPS technique as well as aerial photographs and historical cartography. The technique made it possible to identify shoreline changes and forecast of coastline evolution for more than 100 years [4]. Satellite remote sensing data, having various spectrum ranges, are examined to detect, extract, and monitor coastline changes [5]. Several shoreline mapping techniques on multi-temporal satellite data were compared [6]. Synthetic aperture radar (SAR), capable of all-weather imaging, can acquire images in cloudy and stormy weather as well as day-and-night conditions. The SAR imagery was investigated for mapping and monitoring sediment transport and for improving the quality of land/water body segmentation in order to derive the shorelines [7,8].

A light detection and ranging (LiDAR) system, a scanner system deflecting a laser beam and detecting its reflectance, was applied to detect the spatial patterns and volumetric amounts of coastline erosion along the beach face caused mainly by the tropical storms [9]. The combination of the airborne hyperspectral remote sensing and LiDAR provided a suitable data on the retrieval of the direction and amount of sediment transportation. This made it possible to analyze the dynamics of sandy shorelines and to monitor beach sediments for beach nourishments like the redistribution and the re-sorting of the fill in beach [10]. The unmanned aerial vehicle (UAV) system, a recent popular measuring platform, has increasingly been integrated into many applications. A UAV system was investigated to generate an accurate and valuable geo-information from high-resolution digital imagery at a low cost. The UAV system is an excellent tool for accurate surveys, especially for shallow inland lakes [11]. Accurate 3D representation generated by the UAV system was capable of detecting and visualizing coastal changes over time in order to manage a coastal zone environment [12]. Video cameras, that collect a sequence of images over one tidal cycle, were used to estimate the intertidal beach profile of the nearshore morphology [13]. The images of video cameras were explored to have a strong association with the presence of submerged sandbars through temporal analysis [14]. The video imagery could be utilized to optimize the sandbar estimation without regard to the hydrodynamic data.

Most methodologies focus on detecting and monitoring the coastline without considering the nearshore bathymetry data. The bathymetry data can produce a better understanding on the geomorphic evolution because of its capacity of including the coastlines as a part of its geomorphologic information. Furthermore, the bathymetric data are usually obtained by shipborne sonar sensors: single-beam, multi-beam, and sidescan sonar. A high resolution bathymetry was derived from multi-beam echo-sounders and bathymetric sidescan sonar systems. Due to the bathymetry, it was possible to determine bottom slope corrections and true angles of incidence as well as submarine topography [15]. Multi-beam sonar systems were applied to investigate the seafloor geomorphology from the continental shelf to the shallow-water (more than 10 m deep) coastal zone [16]. A high frequency multi-beam sonar applied to shallow seafloor acquired a bathymetric information that could produce an accurate height and seafloor surface orientation. This is good enough to detect a certain type of seafloor [17]. A sidescan sonar sensor was used to investigate tools for seabed sediments recognition based on accurate sediment data [18]. Thus far, bathymetric sonar is shown as an essential tool to generate seafloor geomorphology. It is impossible to acquire bathymetric data near coastlines with shipborne sonar equipment. This is, because of the depth of a nearshore zone is not deep enough to carry out any operation for a ship. Firstly introduced the gravity-geologic method (GGM) to derive the depth-to-basement of buried bedrock topography in geophysical mapping [19]. Later, satellite altimetry-derived gravity anomalies were adapted to predict bathymetry in the marine environment in several studies [20,21,22,23,24,25]. This is where the shipborne depth measurements are sparsely distributed and limited. A video-based technique has attempted to generate intertidal beach maps from low tide to high tide condition. This aimed to measure the location of the shoreline and monitor the foreshore changes [26]. The coastlines of intertidal beach were represented as horizontal planes as a function of the tidal elevation [27]. A model undertaking proper coastline management was proposed, and an automated procedure for intertidal beach bathymetry was presented using daily video images [28,29]. The limitation of the video-based technique is restricted to only the intertidal coastal areas. 

There is a bathymetric data gap between the shallow seafloor obtained by the shipborne sonar and the intertidal coastal areas by video cameras. For this reason, a bathymetric LiDAR system was introduced. Due to viable laser returns retrieved from the seabed, a bathymetric LiDAR can detect up to sixty meters of depth in clear waters [30]. Furthermore, the LiDAR can provide highly resolved bathymetric data, especially in blue-green wavelength. This is a clear advantage of LiDAR systems in penetrating shallow clear water, generating almost seamless subaerial–submarine bathymetric map [31]. An airborne LiDAR system, developed by the US Army Corps of Engineers, enables monitoring the nearshore bathymetric environments with accurate, densely spaced bathymetric and topographic measurements [32]. A bathymetric LiDAR generates both topographic and bathymetric data to reveal erosional or depositional patterns and geomorphologic changes. This was used to investigate multi-year storm impacts using subaerial and subaqueous sediment volume changes [33]. 

Korea, located on a peninsula in north-eastern Asia, is comprised of western, southern, and eastern coastlines that have different characteristics in terms of their coastal environments. There are big tidal effects and the foreshore is widely distributed in the western coastal zones. Moreover, turbidity is high on western coastal zones due to unclear seawater and many floating materials. Southern coastlines are a typical ria coast and the shape of coastlines is very complicated with numerous islands and rocks. 

This research aims to evaluate the accuracy of a satellite-derived bathymetry estimated by the GGM. This was generated by the combining sparse the Korea Hydrographic and Oceanographic Agency (KHOA) shipborne depth measurements obtained by the multi-beam instruments with a dense satellite altimetry-derived gravity anomalies in shallow waters near the shoreline. Satellite-derived bathymetry grid data were compared with the depth measurements on the KHOA ship tracks and airborne bathymetric LiDAR to assess its accuracy. Finally, this paper will present an improved satellite-derived bathymetry grid data in shallow waters and nearshore coastlines using all depth measurements. This includes the multi-beam and airborne bathymetric LiDAR, and satellite altimetry-derived gravity anomalies in this research.

## 2. Data sets and Methodology

### 2.1. Study Area

**Figure 1:**
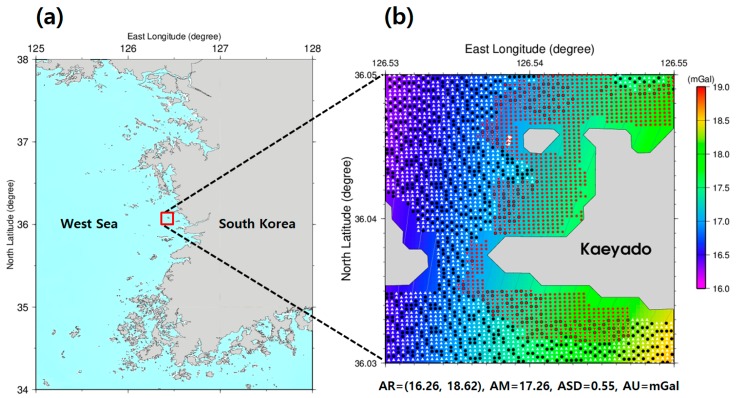
(**a**) Location map of the study area. (**b**) 2292 KHOA multi-beam shipborne locations (white triangles and black circles) and 1201 airborne bathymetric LiDAR locations (red circles); and satellite altimetry-derived gravity anomalies (as a background). AR: amplitude range (=minimum and maximum values); AM: amplitude mean; ASD: amplitude standard deviation; AU: amplitude unit.

This study explores the bathymetry recovery using gravity effects extracted from the depth measurements, including multi-beam and bathymetric LiDAR, and satellite altimetry-derived gravity anomalies over shallow waters offshore Kaeyado Island, located on Ok-do myeon, Kunsan City on the West Sea of Korea. Figure 1 shows the selected study area (126.53°~126.55° E, 36.03°~36.05° N) that is in the shallow waters of the western part of Kaeyado. There are 2292 KHOA multi-beam shipborne locations. The 1528 KHOA multi-beam points are used for control points displayed as white triangles and 764 points are used for checkpoints as black circles. 1201 airborne bathymetric LiDAR points are displayed as red circles. The satellite altimetry-derived gravity anomalies by the Scripps Institution Oceanography [34,35] are displayed by colors.

### 2.2. Data Set

Both the land and the shallow seafloor were surveyed by bathymetric LiDAR and the seafloor measurements were completed by a multi-beam sonar in 2016. The multi-beam sensor is the Seabat 7125 hull mount sonar sensor manufactured by Reson (Slangerup, Denmark). The wavelength of the multi-beam is 400 kHz and the seafloor depth in the study area is less than 30 m. The bathymetric LiDAR sensor is a Coastal Zone Mapping Imaging LiDAR (CZMIL) manufactured by Optech (Vaughan, ON, Canada). The CMZIL uses two wavelengths of 1064 nm and 532 nm. The horizontal coordinates are UTM and its zone is 52. The vertical datum is not the mean sea level (MSL), but the approximate lowest low water (LLW). Table 1 represents the specifications of multi-beam, airborne bathymetric LiDAR, and satellite altimetry-derived gravity anomalies utilized in this study, as shown in Figure 1.

Multi-beam sonar systems are widely used for performing bathymetric surveying. The typical depth of the Seabat 7125 system is 0.5~150 m and the maximum depth is 17 m at 400 kHz. The depth resolution is 6 mm and, the maximum swath depth is 140° in equi-distance mode [35]. The minimum water depth for the sonar system is 1.5 m based on the draft of a ship’s hull and the typical depth of the system. The sonar system, however, typically requires more than 5 m of depth in water for safe operation. Airborne bathymetric LiDAR systems have the advantage of faster and cheaper surveying in shallow water and on coastlines due to a wide swath at aircraft speed. The horizontal and vertical accuracy of the CZMIL system is 3.5 + 0.05 × *d* meter and [0.3^2^ + (0.013 × *d*)^2^]^1/2^ meter, respectively, where *d* is the water depth. The swath width the system is 70 percent of operating altitude. The major limitation of the LiDAR system is turbid and muddy water conditions. The maximum water depth of the LiDAR system depends on the diffuse attenuation coefficient (*K**_d_***). The maximum water depth of shallow and deep channel measurement is 2/*K**_d_*** meter and 4.2/*K**_d_*** meter, respectively [36]. Satellite altimetry-derived gravity anomalies generated by Envisat, CryoSat-2, and Jason-1 satellite altimeters are 1 mGal (=1 × 10^−5^ m/s^2^) accuracy for the global marine gravity field at a 1/2 wavelength spatial resolution of 7 km. The current accuracy of satellite altimetry-derived gravity anomalies is better than 1.7 mGal for latitudes less than 72° and lower accuracy (2~3 mGal) at higher latitudes depending on ice cover [37].

#### 2.2.1. Multi-Beam

Before surveying, several calibrations are required. The first calibration is an offset calibration. There are several sensors like transducers, GPS, gyros as well as multi-beam in a hull mount sonar system, but because the sensors do not coincide with the ship’s center of gravity, accurate offsets between sensors and the center of gravity should be determined. The second one is a multi-beam bar-check. The multi-beam transmits a pan beam that drops a rectangular bar down the transmitter. The distance between the center of gravity and of this type of bar is verified similarly to the distance between the surface water and the bar. The third one is the calibration of the draft mark. This is the distance between the transducer and water surface. The draft mark is mainly dependent on the amount of fuel. The fourth one is the sonar speed calibration in seawater. The speed was approximately 1530 m/s. The last one is the orientation calibration after installing the hull mount multi-beam system. Even tough an error-free surveying system is installed in the wrong directions, the surveying results are incorrect. The orientation of the system is done by calibrating roll, heading, and pitch. 

The Seabat hull mount sonar sensor radiates 240~516 beams in one ping as a pan beam. The surveyed data include the number of pings, data acquisition day/time, the location of data acquisition, vessel motion, gyro data, vessel speed, sonar speed in the sea, tidal data, draft mark and round trip times of the radiated multi-beam. Water depths are calculated by dividing a round trip time by the calibrated sonar speed with its consideration of the tidal effects. The bathymetric data are generated based on a World Geodetic System 1984 (WGS 84) ellipsoid and on a UTM 52 zone. 

#### 2.2.2. Bathymetric LiDAR

LiDAR measurements are made by the round-trip time between the emission of a laser pulse and the arrival of the return signal at the sensor’s receiver. A round-trip distance between the sensor and a target object is determined by multiplying the return signal’s elapsed time by the speed of light. A LiDAR system yields the 3D coordinates of a target object using the distance and the angle of a laser pulse. It is known as a topographic LiDAR. Unfortunately the topographic LiDAR is not suitable for the generation of the seafloor mapping. This is due to most of the laser pulses are returned from the water surface. For this reason, an Optech CZMIL bathymetric LiDAR uses two laser pulses: one is near-IR (1064 nm) laser pulse and the other is a green (532 nm) laser pulse. The former is reflected from the water surface and the latter is from the reflected seafloor topography. Infrared light can be used to detect the water surface location, because infrared light penetrates typical coastal waters very little. The green laser is partially reflected from the water surface and the sea bottom. Distances between the local sea surface and bottom could be calculated by the arrival time differences at the airborne receiver between water surface and sea bottom returns. The water depths at each location in a specific time can be determined by calculating distances between the sea surface and bottom, removing the effects of the wave height, correcting the speed of laser pulses in water, and converting tilted distance to vertical distance using the water nadir angle [38].

The LiDAR system consists of several pieces of equipment: laser, scanner, inertia measurement unit, and GPS. The offsets between a vehicle reference frame and each equipment are accurately calibrated. Scanner angle and boresight are an internal error caused by the movement or impact of the system and has its difficulty to be installed in the LiDAR system at the right directions. Therefore, scanner angle, boresight, roll, heading, and pitch are calibrated. The time interval of GPS is 1 Hz and the distance from the ground GPS reference stations are less than 30 km. The speed of an airplane is 40 knots and the scan angles are less than 20° in consideration of the data accuracy. The scanning pattern of CZMIL is in an elliptical shape. The ground spatial distance is nominally 2 m by 2 m using a deep channel measurement (10 kHz).

As mentioned in Section 2.2, the vertical datum of the multi-beam and LiDAR is different from the satellite altimetry-derived gravity anomalies. The depth from the approximate LLW should be transformed into the depth from the MSL for the next step. A tidebed model is set up from a six adjacent tide observations near the study location, and the calibration value of the depth between the LLW and MSL is decided approximately at 3.585 m. The depth from the MSL is calculated by adding 3.585 cm to the depth from the LiDAR system. The horizontal unit of satellite anomaly data is degree instead of the UTM coordinating system used in LiDAR and the multi-beam system. The Datum Level (DL) of the Kaeyado is 3.585 m below the local MSL provided from the multi-beam system. The multi-beam shipborne bathymetry based on the local MSL was calculated by subtracting 3.585 m from the multi-beam depth based on the DL.

#### 2.2.3. Satellite Altimetry-Derived Gravity Anomaly

The satellite altimetry-derived gravity anomalies were obtained from Scripps Institution of Oceanography (SIO), University of California at San Diego. These were combined to determine a tuning density contrast for an accurate bathymetry prediction from the gravity effects in the off-tracks between ship tracks in shallow waters. The satellite altimetry-derived gravity anomaly data were re-gridded to 1 × 1 arc-seconds by using the “surface” routine, which is a continuous curvature splines, of Generic Mapping Tools (GMT) [39] from the 2 × 2 arc-minutes original data. The satellite altimetry-derived gravity anomalies in the study area showed a change between 16.3 and 18.6 mGal, as illustrated in the background in Figure 1.

### 2.3. Methodology

**Figure 2:**
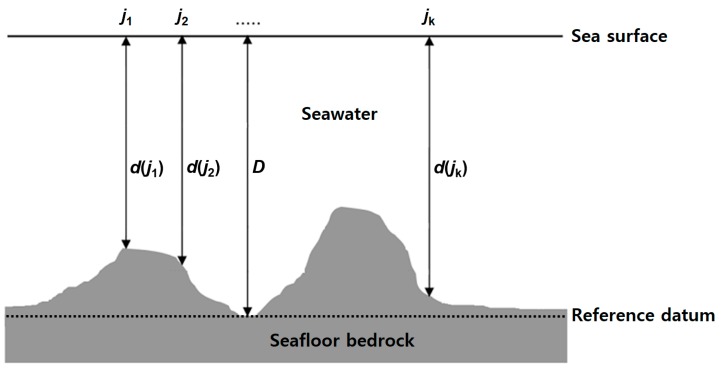
The principle of the gravity geologic method (GGM) to estimate bathymetry (modified from [23]).

In this study, the GGM applied to several studies [20,21,22,23,24,25] to estimate the satellite derived bathymetry was implemented. Figure 2 shows the principles of bathymetry estimation by the GGM using the multi-beam depths of the known *j*-th control points.

The GGM applications to calculate the residual short wavelength gravity from a simple Bouguer term (=2πG(∆*ρ*)) with respect to the reference depth at known *j*-th control points are shown in Figure 2:

*g*_short_(*j*) = 2π*G*(∆*ρ*)(*d*(*j*) − *D*), (1)

where *G* is the gravitational constant, 6.672 × 10^−11^ m^3^ kg^−1^ s^−2^, ∆*ρ* is the density contrast (kg m^−3^), *d*(*j*) is the depth measurement at known *j*-th control points (m), and *D* is the deepest depth at a reference datum (m). The regional long wavelength gravity is generated by subtracting the residual gravity from observed gravity. This was calculated by interpolating the satellite altimetry-derived gravity anomalies at known *j*-th control points.

*g_long_*(*j*) = *g*_obs_(*j*) − *g*_short_(*j*). (2)

The regional long wavelength gravity at unknown *i*-th points were computed by gridding the regional gravity, *g*_long_(*j*), at known *j*-th control points. The residual short wavelength gravity at unknown *i*-th points are calculated from the difference between the observed gravity, *g*_obs_(*i*), and the regional gravity, *g*_long_(*i*):

*g*_short_(*i*) = *g*_obs_(*i*) − *g*_long_(*i*), (3)

where *g*_obs_(*i*) is the satellite altimetry-derived from the gravity anomalies.

Finally, the depth (*d*(*i*)) at unknown *i*-th points were calculated by summing the depth that was extracted by dividing the residual short wavelength gravity at unknown *i*-th points by a simple Bouguer term, and the deepest depth (*D*) as given in Equation (4):

*d*(*i*) = [*g_s_*_hort_(*i*)/2π*G*(∆*ρ*)] + *D*. (4)

In this study, a multi-beam shipborne bathymetry and satellite altimetry-derived gravity anomalies were used to predict the bathymetry in shallow waters around Kaeyado. The multi-beam shipborne bathymetry obtained from the KHOA pertains to the depth with respect to DL. This is the approximate LLW used in a reference surface on a nautical chart. The satellite altimetry-derived gravity anomalies are based on the sea surface, which is approximately the mean sea level. Consequently, the KHOA multi-beam depth measurements were converted to the depths based on the local mean sea level (LMSL) determined from the tidal observations of Kaeyado.

The tuning density contrast between the seawater and the seafloor topography is a very important factor as given in Equation (4) in order to effectively estimate the bathymetry from the short wavelength residual gravity. The estimation of bathymetry using the GGM is influenced by the distribution and number of the depths of known control points, the observed gravity, and the density contrast. The checkpoint method of the GGM was applied to determine a tuning density contrast for accurate bathymetry prediction, because the KHOA multi-beam depth data that have gaps between the shipborne tracks.

In this study, the 2292 KHOA multi-beam depth data were used for predicting the bathymetry by the checkpoints of the GGM. The KHOA multi-beam depth data in the study area were divided as 1528 control points (white triangles in Figure 1) and 764 check points (black circles in Figure 1), respectively, to calculate satellite-derived bathymetry estimation error by the GGM. The control points in the study area were analyzed for the stability of the GGM estimations over a range of density contrasts by the checkpoint method with GGM. 

## 3. Results

**Figure 3:**
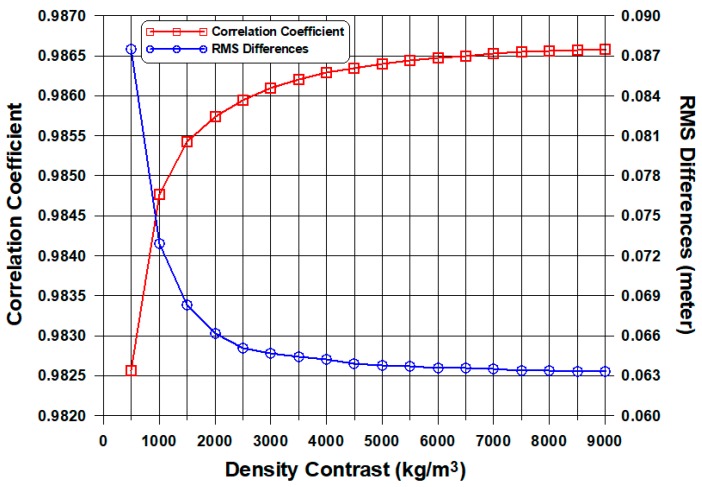
Trade-off diagram for selecting a tuning density contrast of 5000 kg/m^3^ in the study area. RMS: root mean square.

Appropriate tuning density contrast was selected in a trade-off diagram that minimizes root mean square (RMS) errors of the GGM estimations. As shown in Figure 3, the tuning density contrast of about 5000 kg/m^3^ and greater was stabilized in the trade-off diagram, because the RMS errors level off as about 0.064 m along a circle of the blue curve. The tuning density contrast of 5000 kg/m^3^ in this study was chosen to predict satellite-derived bathymetry by the GGM, even though the theoretical density contrast between the seawater and the seafloor topography is 1670 kg/m^3^.

**Figure 4:**
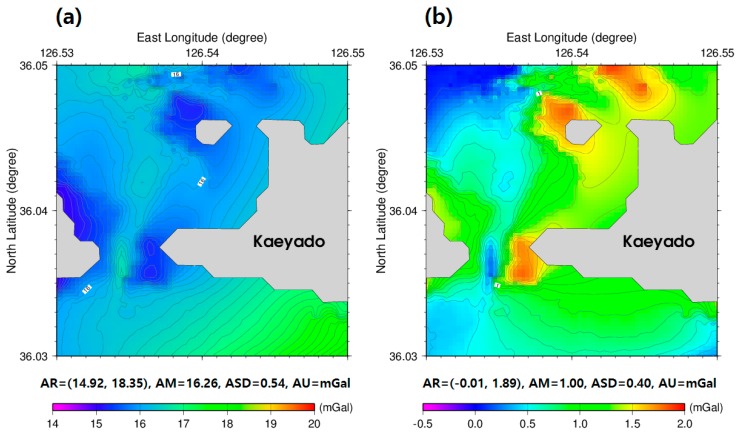
(**a**) 1 arc-seconds regional long wavelength gravity anomalies and (**b**) 1 arc-seconds residual short wavelength gravity anomalies in the study area.

Figure 4a represents 1 arc-seconds gridded regional gravity anomalies computed from Equations (1) and (2). The residual gravity anomalies at unknown *i*-th points are estimated by subtracting the regional gravity anomalies (Figure 4a) from the satellite altimetry-derived gravity anomalies (background in Figure 1b), as illustrated in Figure 4b. These were utilized for the satellite derived bathymetry prediction that applies to the tuning density contrast of 5000 kg/m^3^ determined by the checkpoint method with the GGM in this study.

**Figure 5:**
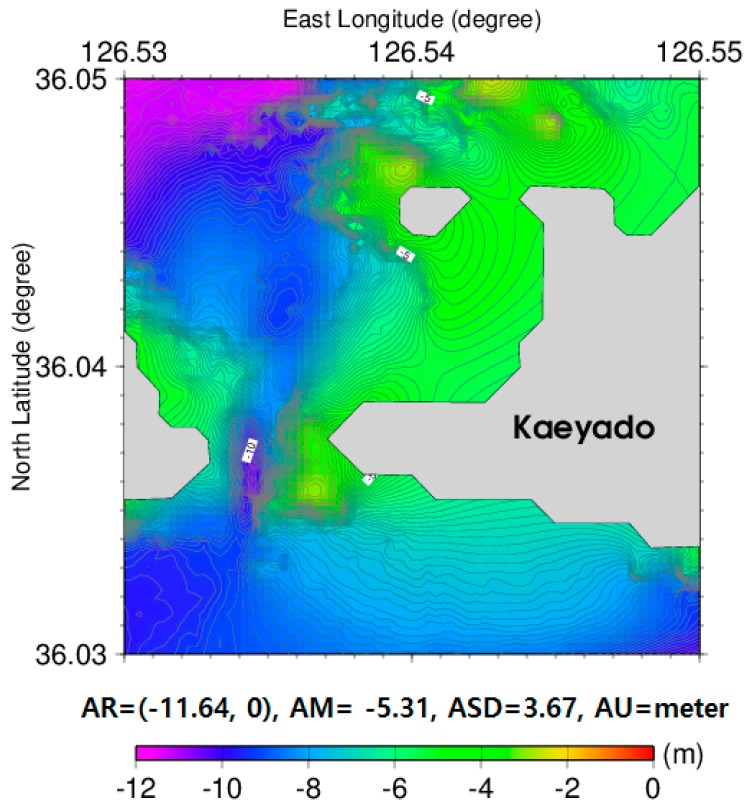
1 arc-seconds satellite-derived bathymetry grid data estimated by the GGM using a tuning density contrast of 5000 kg/m^3^.

The 1 × 1 arc-seconds satellite-derived bathymetry grid data were estimated by the GGM using the tuning density contrast of 5000 kg/m^3^ as shown in Figure 5. Land parts of the islands that includes the Kaeyado in the 1 × 1 arc-seconds satellite derived bathymetry grid data were changed to zero values due to the depth being at a below zero value.

**Figure 6:**
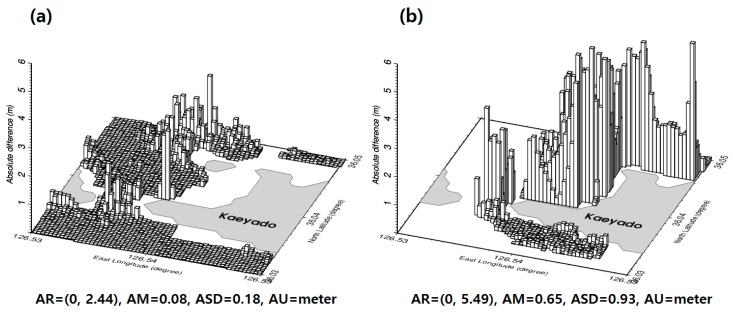
(**a**) Absolute differences of depth between the GGM and KHOA on the 2292 KHOA multi-beam shipborne tracks. (**b**) Absolute differences of depth between the GGM and airborne bathymetric LiDAR on the 1201 airborne bathymetric LiDAR locations.

To evaluate the accuracy of the satellite-derived bathymetry grid data estimated by the GGM, the bathymetry grid was interpolated into the 2292 KHOA multi-beam shipborne locations. These are represented by the white triangles and the black circles in Figure 1. Figure 6a shows the absolute values of depth differences between the satellite-derived bathymetry and the KHOA multi-beam shipborne depths on the 2292 KHOA multi-beam shipborne tracks. As shown in Figure 6a, the statistics of the absolute values of the depth differences are summarized in Table 2.

In addition, the accuracy of satellite-derived bathymetry grid data estimated by the GGM was assessed by extrapolation on airborne bathymetric LiDAR locations that was not used for estimating bathymetry by the GGM. The absolute values of depth differences between satellite-derived bathymetry grid data estimated by the GGM and airborne bathymetric LiDAR were calculated to evaluate the accuracy of the 1201 airborne bathymetric LiDAR locations. These are represented by the red circles in Figure 1. According to the summarized statistics in Table 2, a satellite-derived bathymetry grid data estimated by the GGM in comparison with the airborne bathymetric LiDAR obtained in shallow waters near the shore of the northeastern areas of Kaeyado in Figure 6b shows a maximum difference of 5.49 m. The root mean square error (RMSE) of the absolute depth differences between GGM and KHOA in Table 2 is smaller than that of absolute depth differences between GGM and LiDAR.

**Figure 7:**
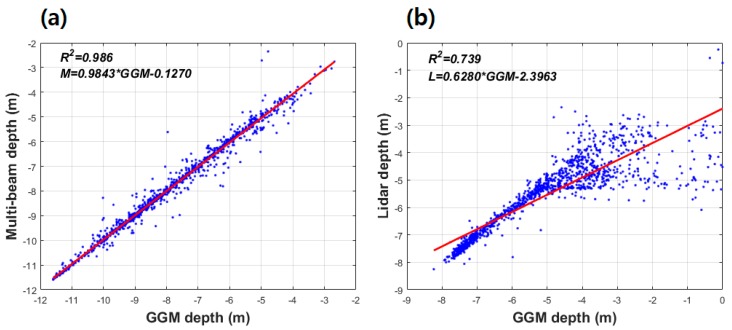
(**a**) The coefficient of determination (*R*^2^) between GGM depth and multi-beam depth and (**b**) the coefficient of determination between GGM depth and airborne bathymetric LiDAR by linear regression.

The coefficient of determination (*R*^2^) was computed to validate the result of correlation between satellite-derived bathymetry by the GGM and KHOA multi-beam depth, and the GGM and airborne bathymetric LiDAR. As shown in Figure 7, GGM depth showed *R*^2^ of 0.986 with KHOA multi-beam depth; whereas, *R*^2^ between GGM depth and airborne bathymetric LiDAR was 0.739. *M*, *L*, and *GGM* in linear regression equation of Figure 7 denote multi-beam depth, airborne bathymetric LiDAR, and satellite-derived bathymetry by the GGM, respectively. These results may indicate that a satellite-derived bathymetry estimated by the GGM in shallow waters is more effective than nearshore.

**Figure 8:**
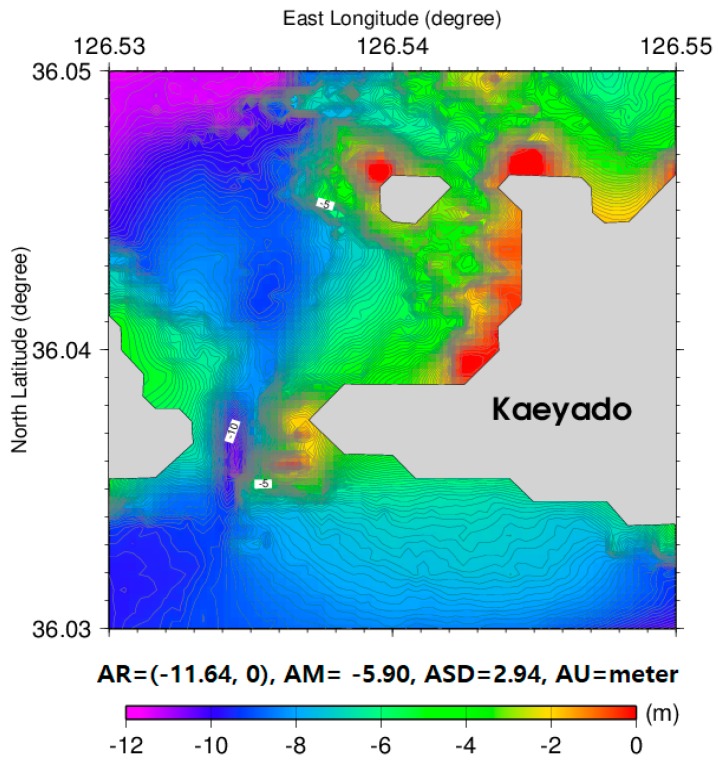
Improved satellite-derived bathymetry estimated by the GGM.

An improved satellite-derived bathymetry estimated by the GGM (IGGM) using all depth measurements that includes a 2292 KHOA multi-beam and 1201 airborne bathymetric LiDAR with a density contrast of 5000 kg/m^3^ was illustrated in Figure 8 as its final result. Since density contrast determined by the GGM is approximately same in small areas, the IGGM was estimated with the assumption that there is no density contrast change. The IGGM of Figure 8 in comparison with the satellite-derived bathymetry estimated by the GGM of Figure 5 was enhanced in very shallow waters near the shore of the upper and left side of Kaeyado.

**Figure 9:**
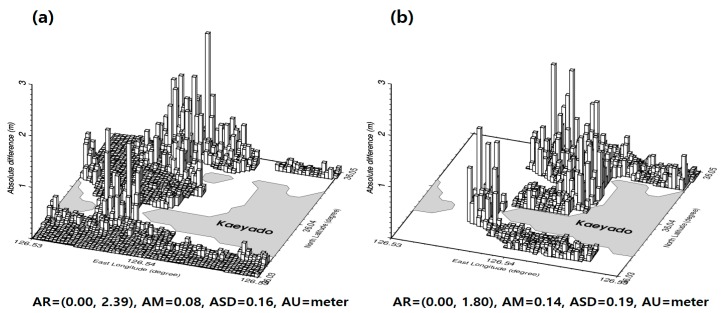
(**a**) Absolute differences of depth between IGGM and KHOA on the 2292 KHOA multi-beam shipborne tracks. (**b**) Absolute differences of depth between IGGM and airborne bathymetric LiDAR on the 1201 airborne bathymetric LiDAR locations.

The IGGM was interpolated into KHOA multi-beam and airborne bathymetric LiDAR locations to compute the absolute differences of the depth for evaluating the accuracy of the improved satellite-derived bathymetry. As shown in Figure 9, the statistics of the absolute values of the depth differences are summarized in Table 3.

The improvement of the RMSE in terms of the absolute depth differences between IGGM and LiDAR in Table 3 is 78% [=(1.13 − 0.24)/1.13] in comparison with that of the absolute depth differences between GGM and LiDAR in Table 2. Whereas the RMSE of the absolute depth differences between IGGM and KHOA in Table 3 is enhanced with about 5% [=[0.19 − 0.18]/0.19], when comparing that of the absolute depth differences between GGM and KHOA in Table 2.

**Figure 10:**
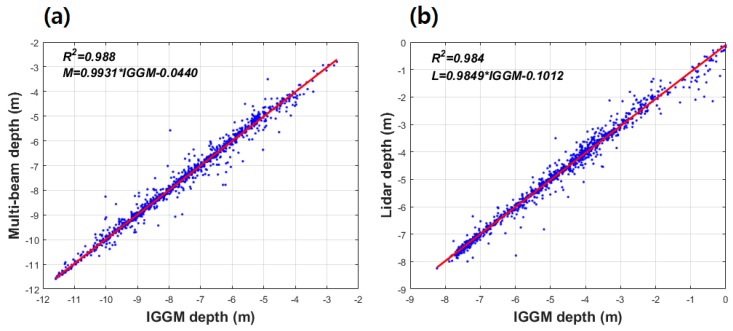
(**a**) The coefficient of determination between IGGM depth and multi-beam depth and (**b**) the coefficient of determination between IGGM depth and airborne bathymetric LiDAR by linear regression.

To compare correlation of between the IGGM and KHOA multi-beam depth, and between the IGGM and airborne bathymetric LiDAR, the coefficient of determination was calculated. The IGGM depth, as shown in Figure 10, showed *R*^2^ of 0.988 and 0.984 with KHOA multi-beam depth and airborne bathymetric LiDAR, respectively. *M*, *L*, and *IGGM* in linear regression equation of Figure 10 denote multi-beam depth, airborne bathymetric LiDAR, and improved satellite-derived bathymetry by the GGM, respectively. It is verified that the IGGM has strong correlation with KHOA multi-beam depth and airborne bathymetric LiDAR. The GGM can be effectively enhanced by combining multi-beam depth in shallow waters and airborne bathymetric LiDAR nearshore coastlines.

**Figure 11:**
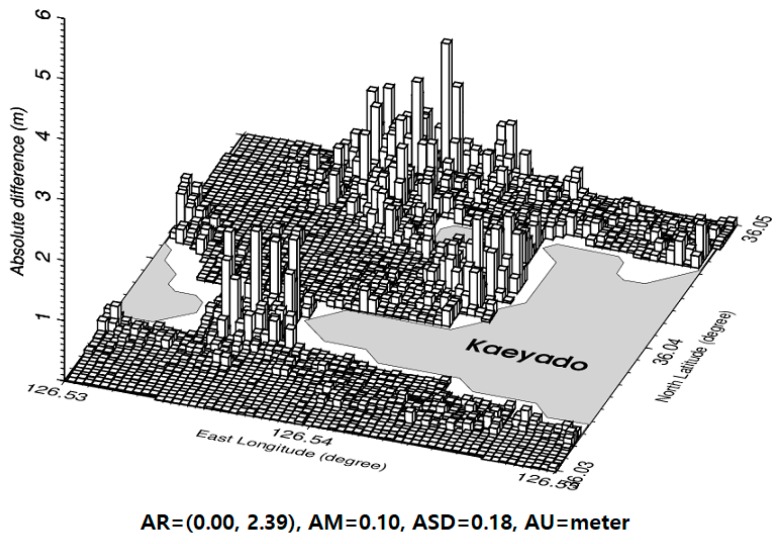
Absolute differences of depth between IGGM and 3493 depth (multi-beam and airborne bathymetric LiDAR) data.

**Figure 12:**
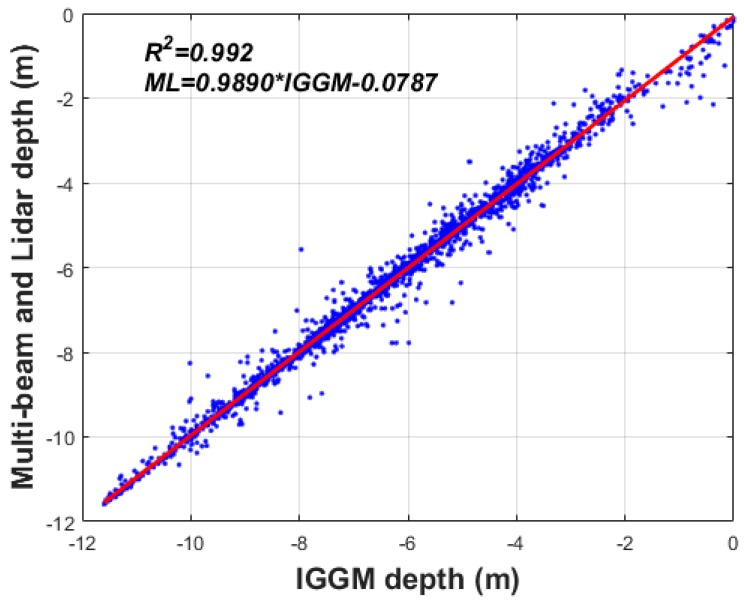
The coefficient of determination between IGGM depth and 3493 depth (multi-beam and airborne bathymetric LiDAR) data by linear regression.

In addition, IGGM was interpolated into 3493 depth measurement locations. These are illustrated in Figure 1 to compute for the absolute differences of the depth for evaluating the accuracy of improved satellite-derived bathymetry. As shown in Figure 11, the minimum, maximum, mean, and standard deviation of the absolute depth differences are 0.00, 2.39, 0.10, and 0.18 m, respectively. The absolute depth differences below 0.20 m (the root mean square error (RMSE) in the statistics of Table 4) in Figure 11 were 86.7%. From small differences between IGGM and 3493 depth (multi-beam and airborne bathymetric LiDAR) data, we concluded that IGGM is enhanced by combining multi-beam depth and airborne bathymetric LiDAR in shallow waters near coastlines. IGGM depth was very highly correlated with 3493 depth (KHOA multi-beam and airborne bathymetric LiDAR) data with *R*^2^ of 0.992, as shown in Figure 12. *ML* and *IGGM* in linear regression equation of Figure 12 denote multi-beam depth and airborne bathymetric LiDAR, and improved satellite-derived bathymetry by the GGM, respectively.

## 4. Discussion

Since 2010, the gravity-geologic method in several studies [20,21,22,23,24] has been applied to predict accurate bathymetry in open sea with single tuning density contrast determined by satellite altimetry-derived gravity anomalies. In this study, multi-beam depth and airborne bathymetric LiDAR were used to enhance satellite-derived bathymetry in shallow waters near coastlines of the western Korea by the gravity-geologic method (GGM). In addition, dense satellite altimetry-derived gravity anomalies were utilized to determine a tuning density of 5000 kg/m^3^ in this study for generating accurate bathymetry by the GGM. According to the results of accuracy evaluation of bathymetry predicted by using only multi-beam depth, the satellite-derived bathymetry grid data had high coefficient of determination of 0.986 with multi-beam depth in shallow waters around Kaeyado Island; whereas, *R*^2^ was 0.739 with airborne bathymetric LiDAR in nearshore zones.

Both multi-beam depth and airborne bathymetric LiDAR were utilized to estimate an IGGM in nearshore zones of the upper and left side of Kaeyado. The IGGM was highly correlated with multi-beam depth and airborne bathymetric LiDAR as 0.988 and 0.984 in *R*^2^, respectively. In the accuracy evaluation of the IGGM, the improvement (78%) of the RMSE of the absolute depth differences between the IGGM and airborne bathymetric LiDAR is larger than that (5%) of the RMSE of the absolute depth differences between the IGGM and multi-beam depth. 

By comparison with the accuracy of bathymetry predicted by only multi-beam depth data in shallow waters around Saemangeum Seawall of the West Sea of Korea in a recent study [25], the satellite-derived bathymetry estimated by combining multi-beam depth and airborne bathymetric LiDAR in this study was remarkably enhanced to the accuracy of 0.2 m in shallow waters near coastlines of the western Korea. Therefore, the gravity-geologic method has an advantage to improve bathymetry accuracy around nearshore by adding airborne bathymetry LiDAR that can observe depth values less than 5 m. This paper concludes that the satellite-derived bathymetry by the GGM can be effectively estimated with newly obtained multi-beam depth and airborne bathymetric LiDAR in shallow waters near coastlines in the future.

## 5. Conclusions

This study evaluated the accuracy of the two satellite-derived bathymetry (GGM and IGGM) estimated by the gravity-geologic method from satellite altimetry-derived gravity anomalies, KHOA multi-beam and airborne bathymetric LiDAR. The bathymetry predicted by the gravity-geologic method was improved by utilizing the topographic effects in the off-tracks computed from the satellite altimetry-derived gravity anomalies, and in the multi-beam and airborne bathymetric LiDAR for shallow waters near the shore of the western Korea. The RMSE of the IGGM was enhanced as accuracy of 0.2 m by using the multi-beam depth and airborne bathymetric LiDAR. This could lead to statistically showing the significant improvement of the shallow waters near coastlines in comparison with that of the GGM. In future studies, the depth data obtained from the airborne bathymetric LiDAR will be applied to enhance the accuracy of bathymetry in shallow waters near the coastlines.

## Figures and Tables

**Table 1:** The specification of multi-beam, airborne bathymetric light detection and ranging (LiDAR), and satellite altimetry-derived gravity anomalies. CZMIL: Coastal Zone Mapping Imaging LiDAR; WGS: World Geodetic System.

Type	Multi-Beam Bathymetry	Airborne Bathymetric LiDAR	Satellite Altimetry-Derived Gravity Anomalies
Sensor	Seabat 7125	CZMIL	Altimeter
Platform	shipborne	airborne	spaceborne
Ellipsoid	WGS-84	WGS-84	WGS-84
Unit	meter	meter	mGal
Reference surface	Datum Level	Datum Level	Mean Sea Level

**Table 2:** Statistics of absolute values of depth differences between GGM and Korea Hydrographic and Oceanographic Agency (KHOA) multi-beam and between GGM and bathymetric LiDAR (Unit: meter).

	Min.	Max.	Mean	Std. Dev.	RMSE
GGM–KHOA	0.00	2.44	0.08	0.18	0.19
GGM–LiDAR	0.00	5.49	0.65	0.93	1.13

**Table 3:** Statistics of absolute values of depth differences between the improved GGM (IGGM) and KHOA multi-beam and between the IGGM and bathymetric LiDAR (Unit: meter).

	Min.	Max.	Mean	Std. Dev.	RMSE
IGGM–KHOA	0.00	2.39	0.08	0.16	0.18
IGGM–LiDAR	0.00	1.80	0.14	0.19	0.24

**Table 4:** Statistics of absolute values of depth differences between IGGM and 3493 (KHOA multi-beam and airborne bathymetric LiDAR) depth locations (Unit: meter).

	Min.	Max.	Mean	Std. Dev.	RMSE
IGGM − (KHOA + LiDAR)	0.00	2.39	0.10	0.18	0.20
